# GATA6 promotes epithelial-mesenchymal transition and metastasis through MUC1/*β*-catenin pathway in cholangiocarcinoma

**DOI:** 10.1038/s41419-020-03070-z

**Published:** 2020-10-15

**Authors:** Xiang Deng, Peng Jiang, Jian Chen, Jianwei Li, Dajiang Li, Yu He, Yan Jiang, Yujun Zhang, Shuangnian Xu, Xi Li, Shuguang Wang, Feng Tian

**Affiliations:** 1 Department of Hepatobiliary Surgery, Southwest Hospital, Army Medical University (Third Military Medical University), Chongqing, 400038 China; 2 Department of Abdominal Surgery, Guiqian International General Hospital, Guiyang, 550000 Guizhou China; 3 Department of Hematology, Southwest Hospital, Army Medical University (Third Military Medical University), Chongqing, 400038 China

**Keywords:** Bile duct cancer, Metastasis

## Abstract

GATA6 acts as an oncogene or tumour suppressor in different cancers. Previously, we found that aberrant expression of GATA6 promoted metastasis in cholangiocarcinoma (CCA). However, the mechanism by which GATA6 promotes metastasis in CCA is unclear. In the present study, we aimed to investigate the role of GATA6 in CCA cell epithelial–mesenchymal transition (EMT). Our results showed that GATA6 expression was positively associated with N-cadherin and vimentin expression but negatively associated with E-cadherin expression in 91 CCA samples. GATA6 promoted EMT and metastasis in CCA cells in vitro and in vivo based on knockdown and overexpression analyses. ChIP-sequencing data revealed that MUC1 is a novel downstream target of GATA6. GATA6 upregulated MUC1 expression through binding to both the 1584 and 1456 GATA-motifs in the promoter region and enhancing its transcription by luciferase reporter assays and point-mutant assays. MUC1 expression was positively associated with N-cadherin and vimentin expression but negatively associated with E-cadherin expression in 91 CCA samples. In addition, MUC1 promoted EMT in CCA cells based on knockdown and overexpression analyses. Moreover, MUC1 knockdown significantly abrogated the GATA6-induced EMT in CCA cells, indicating that MUC1 promoted EMT through upregulating MUC1 in CCA cells. *β*-Catenin is a putative transcriptional coactivator that regulates EMT in cancers. Our data showed that MUC1 expression was positively associated with nuclear *β*-catenin expression in 91 CCA samples. MUC1 upregulated nuclear *β*-catenin expression in CCA cells. Moreover, MUC1 bound to *β*-catenin in CCA cells based on protein immunoprecipitation analyses. MUC1 knockdown significantly decreased the binding of MUC1 to *β*-catenin, and thereby decreased nuclear *β*-catenin protein levels in CCA cells, indicating that MUC1 bound to *β*-catenin and increased its nuclear expression in CCA cells. Together, our results show that GATA6 promotes EMT through MUC1/*β*-catenin pathway in CCA, indicating potential implications for anti-metastatic therapy.

## Background

The incidence of cholangiocarcinoma (CCA) has progressively increased worldwide over the past four decades^1^. Surgery is the only option for radical treatment. Unfortunately, ~65% of patients with CCA are ineligible for radical surgery because of tumour invasion and metastasis^1,2^. Thus, understanding the molecular mechanisms of CCA metastasis is currently an important issue.

GATA-binding protein 6 (GATA6) belongs to an evolutionarily conserved family that regulates gene expression through binding to the GATA motif in the promoter region^3^. It has been reported that GATA6 plays different roles in different cancer types. GATA6 promotes tumour progression and acts as an oncoprotein in gastric cancer, colorectal cancer, breast cancer and cutaneous T-cell lymphoma^4–7^, but it suppresses tumour progression in astrocytoma and hepatocellular carcinoma^8,9^. Moreover, the functions of GATA6 in pancreatic cancer and ovarian cancer are controversial^10–13^. Our previous study showed that aberrant expression of GATA6 promotes invasion and metastasis in CCA^14,15^. However, the underlying mechanisms by which GATA6 promotes CCA metastasis are not well known.

During the past few years, the role of the epithelial to mesenchymal transition (EMT) in tumour progression has attracted more attention^16^. EMT is a reversible dynamic process during which cancer cells gradually lose the epithelial phenotype and adopt mesenchymal characteristics. EMT is an early event of cancer cell dissemination from the primary tumour, which plays prominent roles in CCA metastasis^17^. GATA6 is reported to act as a conserved inducer of the endodermal EMT in embryonic development^18^. However, the role of GATA6 in EMT of CCA cells is completely unknown.

In the present study, we evaluated the function of GATA6 in the EMT of CCA cells through in vitro and in vivo experiments. Moreover, we identified MUC1 as a novel target of GATA6 and found that GATA6 promoted EMT through upregulating MUC1. In addition, we investigated the role of MUC1 in regulating unclear *β*-catenin expression in CCA cells. Our present findings reveal important roles of GATA6 in EMT and metastasis of CCA cells, and provide potential implications for anti-metastatic therapy.

## Materials and methods

### CCA patients and clinical samples

Ninety-one CCA patients who underwent surgical resection between January 2008 and December 2014 in our centre were included. Ninety-one cancer samples and 31 matched paracancerous samples were embedded in paraffin and used for immunohistochemistry (IHC). Overall survival was calculated from the date of surgery until the date of last contact. The recurrence time was defined as the interval from the date of surgery to the date of tumour recurrence. Informed consent was obtained from all patients prior to specimen and data collection.

### Immunohistochemistry

The expression of GATA6, MUC1, *β*-catenin and EMT markers (E-cadherin, N-cadherin and vimentin) in CCA samples was detected using IHC. IHC was performed using the method described in our previous study^14^. Antibodies against GATA6 (1 : 200, Abcam, Cambridge, MA, USA), MUC1 (1 : 500, Abcam, Cambridge, MA, USA), *β*-catenin (1 : 100, Proteintech, Wuhan, China), E-cadherin (1 : 100, Proteintech, Wuhan, China), N-cadherin (1 : 100, Proteintech, Wuhan, China) and vimentin (1 : 100, Proteintech, Wuhan, China) were used. Two independent investigators assessed the percentage and staining intensity of the positive cancerous cells.

### CCA cell lines and transfection

Two CCA cell lines, QBC939 and RBE, were used in the present study, consistent with our previous studies^14,15^. Cells were transfected with lentivirus vectors encoding short hairpin RNA targeting human GATA6 for GATA6 knockdown or empty lentivirus vectors for control (Control) (GenePharma, Shanghai, China). The coding sequence (CDS) template of GATA6 was synthesized chemically and cells were transfected with lentivirus vectors encoding GATA6 CDS DNA for GATA6 overexpression (exGATA6) (GenePharma, Shanghai, China). In addition, cells were transfected with lentivirus vectors encoding short hairpin RNA targeting MUC1 for MUC1 knockdown or MUC1 CDS DNA for MUC1 overexpression (exGATA6) (GenePharma, Shanghai, China).

### Xenotransplantation of CCA cells in nude mice

The xenotransplantation mouse model was generated as described in our previous study^14^. Four-week-old male nude mice were used (Laboratory Animal Centre, Third Military Medical University). Each group contained five mice. Each mouse was anaesthetized and the spleen was carefully exteriorized through a peritoneal incision by using forceps in the animal operation room of our lab. Approximately 5 × 10^5^ CCA cells were injected into the spleen. Then, the spleen was placed back in the abdominal cavity and the abdominal muscle and skin were sutured. All experiments were conducted in accordance with the Public Health Service Policy on Humane Care and Use of Laboratory Animals. Eight weeks later, mice were observed by in vivo bioluminescence and small animal magnetic resonance imaging (MRI). Mice were killed to confirm liver metastases at autopsy. The liver metastasis volume was calculated using the equation reported in a previous study^19^: *V*^Liver metastases^ = SUM (*V*^Each mass^), *V*^Each mass^ = (length × width^2^)/2. Liver metastases were also embedded in paraffin for haematoxylin and eosin staining. The expression of GATA6, MUC1, *β*-catenin and EMT markers in liver metastases were detected using real-time PCR and western blot (WB) analyses.

### Chromatin immunoprecipitation and ChIP sequencing

Chromatin immunoprecipitation (ChIP) was performed using the SimpleChIP^®^ Plus Sonication ChIP kit (Cell Signaling Technology, Danvers, MA, USA) according to the manufacturer’s protocol. An anti-GATA6 antibody (1 : 50, Cell Signaling Technology, Danvers, MA, USA) was used. GATA6-ChIP DNA and the input DNA of CCA cells were used for ChIP sequencing (ChIP-Seq) analyses (Aksomics, Shanghai, China). A region with a *P*-value (−10*log) ≥ 50 was defined as a GATA6-enriched region. The primers used are listed in Supplementary Table S1.

### Luciferase reporter activity

CCA cells were transfected with luciferase reporter vectors containing the wild-type or point-mutant MUC1 promoter (2500 bp, GenePharma, Shanghai, China). The pRL *Renilla* luciferase reporter vectors (Promega, Madison, WI, USA) were transfected for normalization and transfection efficiency control. Transfections were performed using Lipofectamine 3000 (Invitrogen, Carlsbad, CA, USA) according to the manufacturer’s instructions. After 36 h, cells were lysed with passive lysis buffer using the luciferase kit (Promega, Madison, WI, USA). The reporter activities were determined using the Dual-Glo Luciferase Assay system (Promega, Madison, WI, USA). The experiments were repeated independently three times.

### Protein immunoprecipitation

The proteins extracted from CCA cells were incubated with the rabbit monoclonal anti-MUC1 antibody (Abcam, Cambridge, MA, USA) or rabbit polyclonal anti-*β*-catenin antibody (Proteintech, Wuhan, China) overnight at 4 °C with constant rotation. The immunocomplexes were captured by Protein A agarose (Invitrogen, Carlsbad, CA, USA) for 1 h at 4 °C with constant rotation. Then, the immunocomplexes were analysed by WB. The experiments were repeated independently three times.

The protocols used for real-time PCR, WB, wound-healing assay (migration) and Transwell assay (invasion) are provided in Document S1.

### Statistical analysis

Data were analysed by using SPSS 17.0 or SAS 9.2 software. Continuous data were analysed using the *t*-test. The *χ*^2^-analysis or Fisher’s exact test was used for categorical data. Spearman’s correlation analyses were performed. Kaplan–Meier and Cox analyses were applied to assess overall survival and recurrence-free survival. Statistical significance was set to *P* < 0.05.

## Results

### GATA6 promotes EMT in CCA

**Fig. 1:**
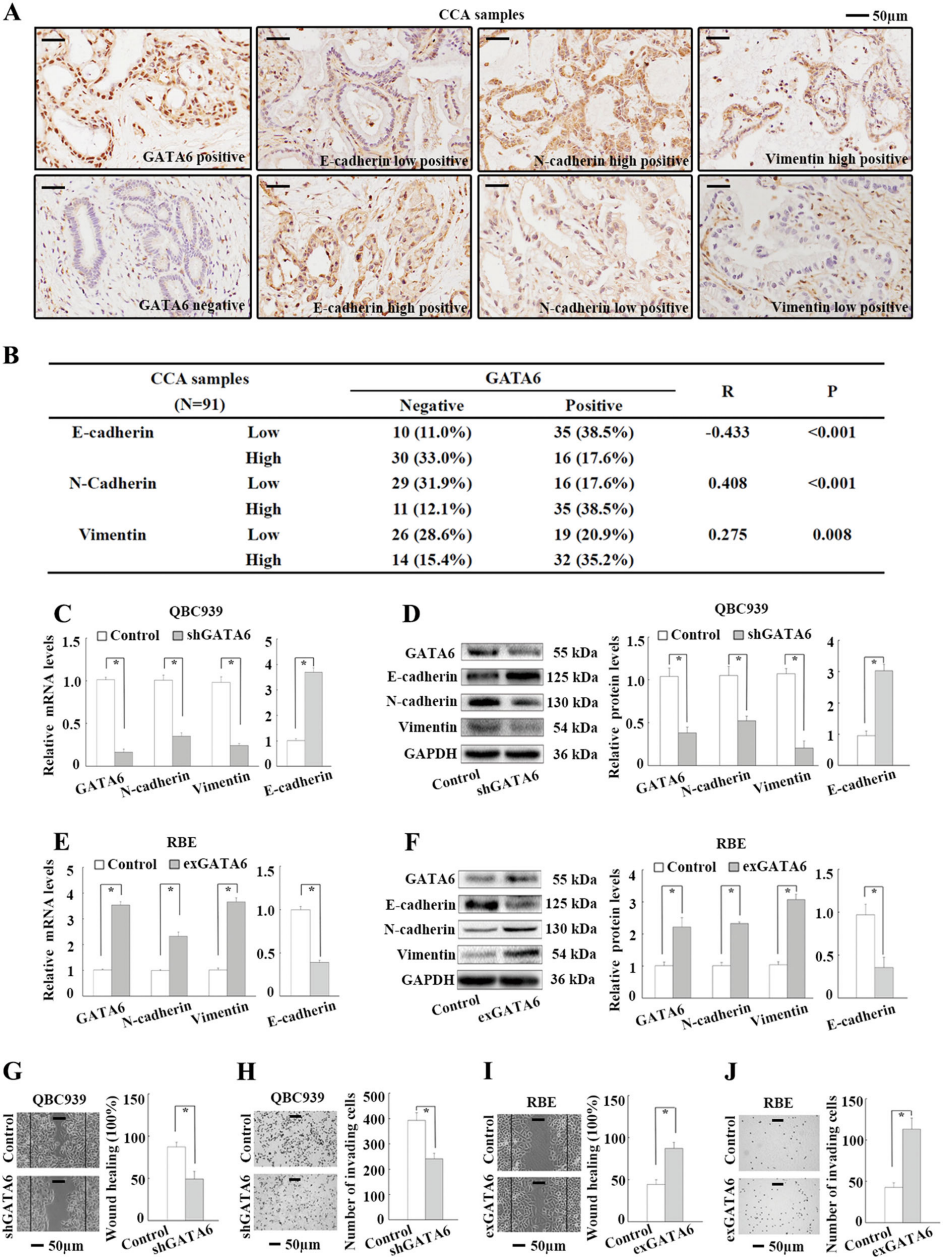
GATA6 promotes EMT in CCA. **a** IHC staining of GATA6 and EMT markers (E-cadherin, N-cadherin and vimentin) in CCA samples. **b** Correlations between GATA6 and EMT markers in 91 CCA samples by Spearman’s correlation analyses. **c** Impact of GATA6 knockdown on EMT marker mRNA levels in QBC939 cells by real-time PCR analyses. **d** Impact of GATA6 knockdown on EMT marker protein levels in QBC939 cells by WB analyses. **e** Impact of GATA6 overexpression on EMT marker mRNA levels in RBE cells by real-time PCR analyses. **f** Impact of GATA6 overexpression on EMT marker protein levels in RBE cells by WB analyses. **g** Impact of GATA6 knockdown on QBC939 cell migration by wound-healing analyses. **h** Impact of GATA6 knockdown on QBC939 cell invasion by transwell analyses. **i** Impact of GATA6 overexpression on RBE cell migration by wound-healing analyses. **j** Impact of GATA6 overexpression on RBE cell invasion by transwell analyses. Control: cells transfected with empty lentivirus vectors. shGATA6: cells transfected with lentivirus vectors encoding short hairpin RNA targeting human GATA6. exGATA6: cells transfected with lentivirus vectors encoding human GATA6 CDS DNA. *P < 0.05.

To explore the role of GATA6 in the EMT of CCA cells, we first observed the expression of GATA6 and EMT markers in 91 cancer samples and 31 matched paracancerous samples by IHC. GATA6 was expressed in the nucleus with scattered signals detected in the cytoplasm (Fig. 1a). The cancer samples (or paracancerous samples) were considered GATA6-positive if more than 5% of the cancer cells (or biliary epithelial cells) showed GATA6-positive nuclear staining. Fifty-one cancer samples (56.0%) showed GATA6-positive expression, whereas all 31 paracancerous samples were negative for GATA6 (Supplementary Fig. S1). GATA6 expression was significantly associated with lymph node metastasis in 91 patients with CCA (Supplementary Table S2). Moreover, patients with GATA6 expression showed significantly poorer overall survival and earlier recurrence than patients without GATA6 expression according to the Kaplan–Meier analyses (Supplementary Fig. S2). The expression of E-cadherin, N-cadherin or vimentin in CCA samples was separated into high and low groups according to the median percentage of stained cancer cells (Fig. 1a). Spearman’s correlation analyses revealed positive correlations of GATA6 expression with N-cadherin and vimentin expression, but a negative correlation with E-cadherin expression in the 91 CCA samples (Fig. 1b). Second, the role of GATA6 in EMT was investigated in two CCA cell lines, QBC939 and RBE. GATA6 knockdown in QBC939 cells significantly upregulated E-cadherin mRNA expression but downregulated N-cadherin and vimentin mRNA expression based on real-time PCR analyses (Fig. 1c). Consistent with these findings, WB analyses revealed significantly increased protein levels of E-cadherin and decreased protein levels of N-cadherin and vimentin following GATA6 knockdown in QBC939 cells (Fig. 1d). Following GATA6 overexpression in RBE cells, the mRNA and protein levels of E-cadherin were reduced, whereas those of N-cadherin and vimentin were significantly increased (Fig. 1e, f). In addition, GATA6 knockdown significantly decreased QBC939 cell migration and invasion (Fig. 1g, h), and GATA6 overexpression significantly increased RBE cell migration and invasion (Fig. 1i, j). Together, these results suggest that GATA6 promotes EMT in CCA.

### *MUC1* is a novel target gene of GATA6 in CCA cells

**Fig. 2:**
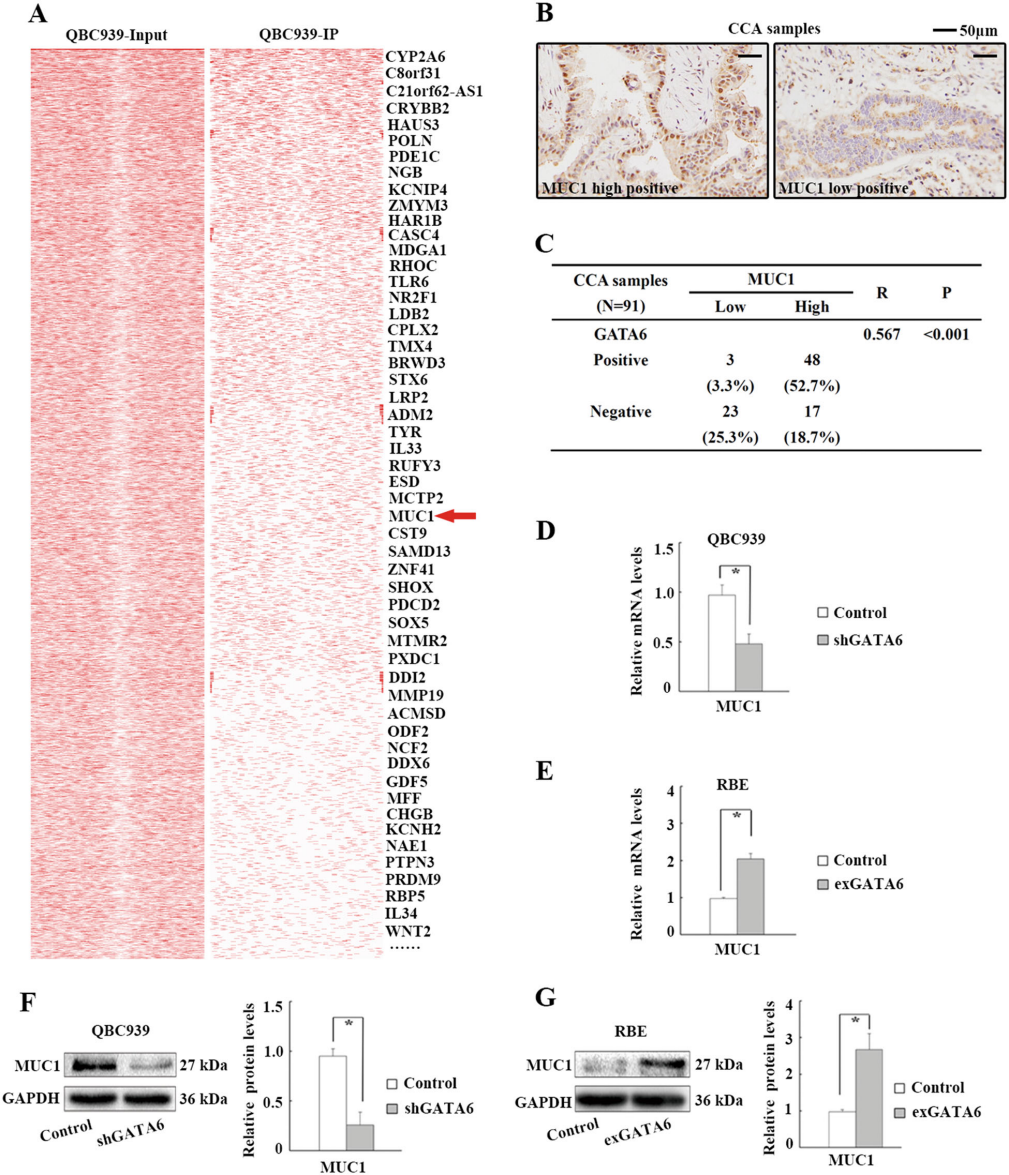
GATA6 upregulates MUC1 expression in CCA cells. **a** GATA6-enriched genes in QBC939 cells by ChIP-Seq analyses. **b** IHC staining of MUC1 in CCA samples. **c** Correlations between GATA6 and MUC1 expression in 91 CCA samples by Spearman’s correlation analyses. **d** Impact of GATA6 knockdown on MUC1 mRNA levels in QBC939 cells by real-time PCR analyses. **e** Impact of GATA6 overexpression on MUC1 mRNA levels in RBE cells by real-time PCR analyses. **f** Impact of GATA6 knockdown on MUC1 protein levels in QBC939 cells by WB analyses. **g** Impact of GATA6 overexpression on MUC1 protein levels in RBE cells by WB analyses. Control: cells transfected with empty lentivirus vectors. shGATA6: cells transfected with lentivirus vectors encoding short hairpin RNA targeting human GATA6. exGATA6: cells transfected with lentivirus vectors encoding human GATA6 CDS DNA. *P < 0.05.

The mechanism by which GATA6 promoted the EMT of CCA cells was investigated. As GATA6 mainly regulates gene expression by binding to the GATA motif in the promoter region^20^, we used ChIP-Seq analysis to investigate the genome-wide distribution of GATA6 in QBC939 cells. Peak detection data defined 9368 GATA6-enriched regions (*P*-value (−10*log) ≥ 50). A total of 470 GATA6-enriched regions were located in the promoters of various genes and 157 GATA6-enriched genes were defined (fold-enrichment ≥ 5) (Supplementary Table S3). EMT markers were not included in the GATA6-enriched genes, indicating that GATA6 regulates the expression of EMT markers through other proteins. Then, we screened the GATA6-enriched genes and finally focused on a new potential candidate, MUC1, based on the following evidence: (i) MUC1 is a glycoprotein that induces metastasis and the EMT in cancers^21–23^; (ii) overexpression of MUC1 promotes metastasis in CCA^24^; and (iii) our ChIP-Seq data showed that GATA6 was enriched in the MUC1 promoter region in CCA cells (Fig. 2a). To validate the role of GATA6 in MUC1 expression, we first observed the correlation between MUC1 and GATA6 expression in CCA samples. All 91 CCA samples showed membrane, cytoplasmic and/or nuclear MUC1 expression in CCA cells (Fig. 2b). Sixty-five patients were classified as having high MUC1 expression, with ≥50% of CCA cells showing positive staining. Spearman’s correlation analyses revealed a significant positive correlation between MUC1 and GATA6 expression (Fig. 2c). Second, the role of GATA6 in MUC1 expression was investigated in CCA cells. The levels of MUC1 mRNA were significantly downregulated by GATA6 knockdown and upregulated by GATA6 overexpression in CCA cells (Fig. 2d, e). Consistent with these findings, the protein levels of MUC1 were also regulated by GATA6 in CCA cells (Fig. 2f, g). Together, the results show that MUC1 is a novel downstream gene regulated by GATA6 in CCA.

### GATA6 upregulates MUC1 expression by enhancing its transcriptional activity through binding to both 1584 and 1456 GATA motifs in the promoter

**Fig. 3:**
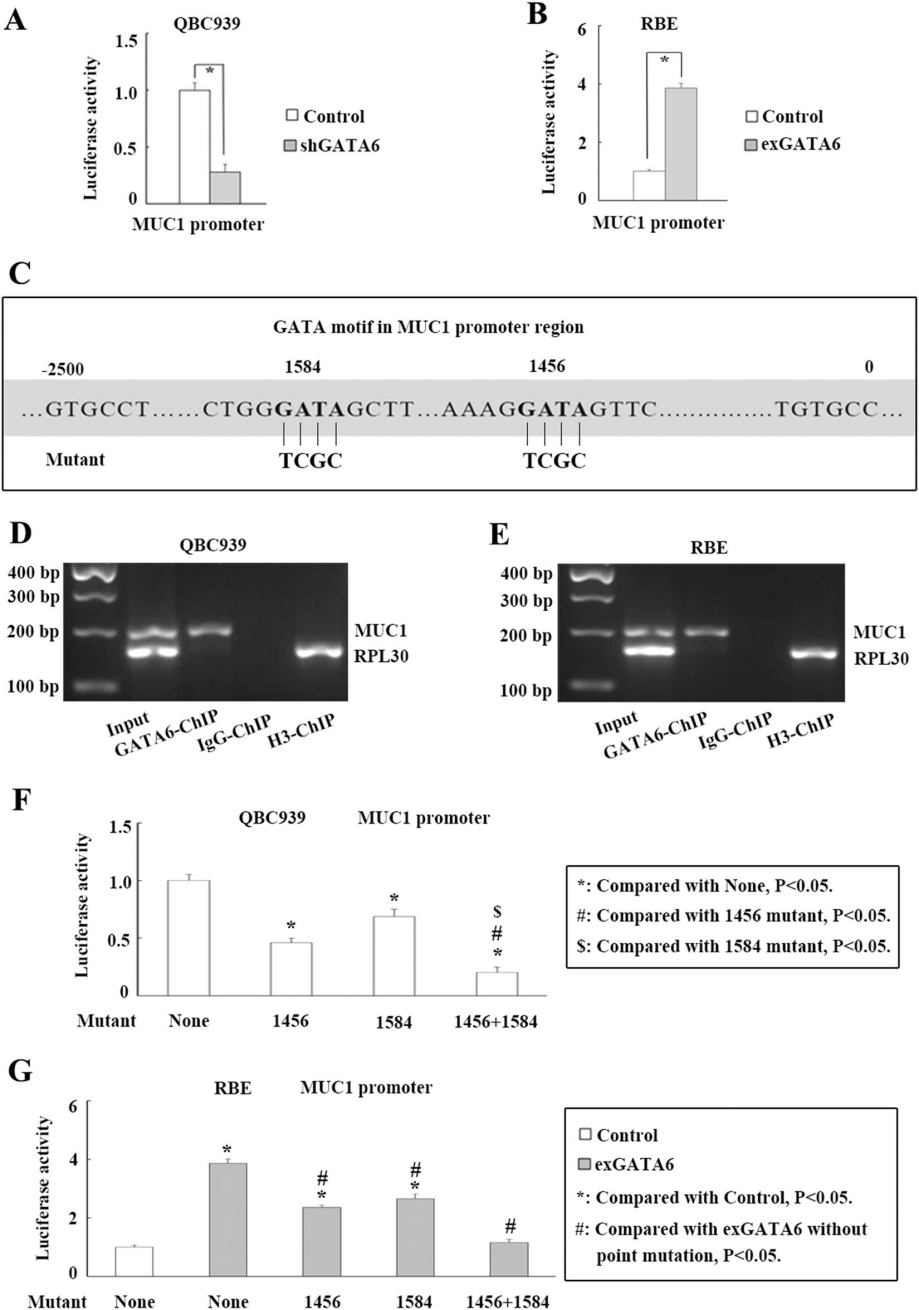
GATA6 upregulates MUC1 expression by binding to both 1584 and 1456 GATA motifs in the promoter and enhancing its transcription. **a** Impact of GATA6 knockdown on MUC1 promoter activity in QBC939 cells by luciferase reporter analyses. **b** Impact of GATA6 overexpression on MUC1 promoter activity in RBE cells by luciferase reporter analyses. **c** Bioinformatic data show two GATA motifs (1584 and 1456) in MUC1 promoter region. **d**, **e** GATA6 bound to MUC1 promoter in QBC939 and RBE cells by ChIP analyses. **f** Impact of 1584 or (and) 1456 GATA motif mutation on GATA6 regulating MUC1 promoter activity in QBC939 cells. **g** Impact of 1584 or (and) 1456 GATA motif mutation on GATA6 overexpression regulating MUC1 promoter activity in RBE cells. Control: cells transfected with empty lentivirus vectors. shGATA6: cells transfected with lentivirus vectors encoding short hairpin RNA targeting human GATA6. exGATA6: cells transfected with lentivirus vectors encoding human GATA6 CDS DNA. *P < 0.05.

Next, the molecular mechanism by which GATA6 upregulates MUC1 expression was investigated. According to luciferase reporter analyses, GATA6 knockdown significantly reduced MUC1 promoter activity in QBC939 cells (Fig. 3a), whereas MUC1 promoter activity was significantly increased following GATA6 overexpression in RBE cells (Fig. 3b). Two GATA motifs (1456 and 1584) were identified in the MUC1 promoter (Fig. 3c). ChIP data showed that GATA6 bound to MUC1 promoter in QBC939 and RBE cells (Fig. 3d, e). Then, we conducted luciferase reporter analyses of GATA-motif mutants to identify the site at which GATA6 enhances MUC1 transcription. Mutation of the 1456 or 1584 motif partially abolished the ability of GATA6 to increase MUC1 promoter activity in QBC939 cells (Fig. 3f). Mutation of both sites (1584 and 1456 motifs) completely abolished the ability of GATA6 to increase MUC1 promoter activity (Fig. 3f). Consistent with these findings, mutation of the 1456 or 1584 motif partially abolished the ability of GATA6 overexpression to increase MUC1 promoter activity and mutation of both sites (1584 and 1456 motifs) completely abolished the ability of GATA6 overexpression to increase MUC1 promoter activity in RBE cells (Fig. 3g). Together, the results show that GATA6 upregulates MUC1 by enhancing its transcriptional activity through binding to both the 1584 and 1456 GATA motifs in the promoter.

### GATA6 promotes EMT through upregulating MUC1 in CCA cells

**Fig. 4:**
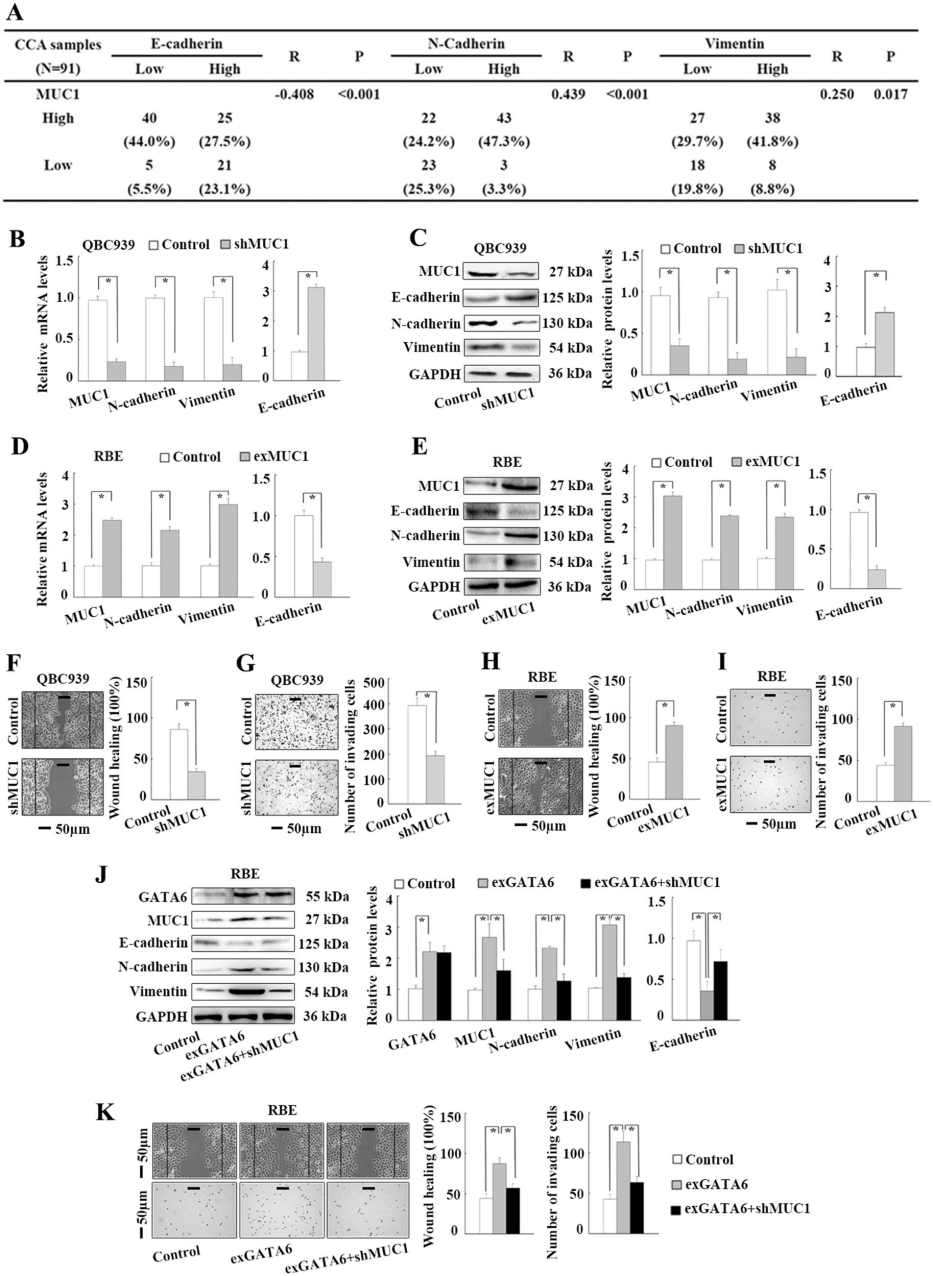
GATA6 promotes EMT through upregulating MUC1 in CCA cells. **a** Correlations between MUC1 and EMT markers in 91 CCA samples by Spearman’s correlation analyses. **b** Impact of MUC1 knockdown on EMT marker mRNA levels in QBC939 cells by real-time PCR analyses. **c** Impact of MUC1 knockdown on EMT marker protein levels in QBC939 cells by WB analyses. **d** Impact of MUC1 overexpression on EMT marker mRNA levels in RBE cells by real-time PCR analyses. **e** Impact of MUC1 overexpression on EMT marker protein levels in RBE cells by WB analyses. **f** Impact of MUC1 knockdown on QBC939 cell migration by wound-healing analyses. **g** Impact of MUC1 knockdown on QBC939 cell invasion by transwell analyses. **h** Impact of MUC1 overexpression on RBE cell migration by wound-healing analyses. **i** Impact of MUC1 overexpression on RBE cell invasion by transwell analyses. **j** MUC1 knockdown significantly abrogated GATA6 overexpression induced EMT marker protein expression in RBE cells. **k** MUC1 knockdown significantly abrogated GATA6 overexpression induced RBE cell migration and invasion by wound-healing and tranwell analyses. Control: cells transfected with empty lentivirus vectors. shMUC1: cells transfected with lentivirus vectors encoding short hairpin RNA targeting human MUC1. exMUC1: cells transfected with lentivirus vectors encoding human MUC1 CDS DNA. exGATA6: cells transfected with lentivirus vectors encoding human GATA6 CDS DNA. *P < 0.05.

To explore whether GATA6 promoted EMT through upregulating MUC1, we first validated the role of MUC1 in the EMT of CCA cells. In the 91 CCA samples, MUC1 expression was significantly negatively associated with E-cadherin expression but positively associated with N-cadherin and vimentin expression according to Spearman-correlation analyses (Fig. 4a). High MUC1 expression was significantly associated with low histological grade and lymph node metastasis (Supplementary Table S2). In addition, following MUC1 knockdown in QBC939 cells, the mRNA and protein levels of E-cadherin were significantly increased and those of N-cadherin and vimentin were reduced based on real-time PCR and WB (Fig. 4b, c). Conversely, following MUC1 overexpression in RBE cells, the mRNA and protein levels of E-cadherin were reduced, and those of N-cadherin and vimentin were significantly increased (Fig. 4d, e). In addition, QBC939 cell migration and invasion were significantly suppressed by MUC1 knockdown (Fig. 4f, g), and RBE cell migration and invasion were significantly promoted by MUC1 overexpression (Fig. 4h, i). The above data indicated that MUC1 promoted EMT in CCA. Next, MUC1 knockdown was performed in GATA6-overexpressing RBE cells to block the GATA6-induced upregulation of MUC1. GATA6-induced downregulation of E-cadherin and upregulation of N-cadherin and vimentin were significantly abrogated by MUC1 knockdown in RBE cells (Fig. 4j). Consistent with these findings, the GATA6-induced increases in RBE cell migration and invasion were significantly abrogated by MUC1 knockdown (Fig. 4k). Together, these results suggest that GATA6 promotes EMT through upregulating MUC1 in CCA cells.

### MUC1 binds to *β*-catenin and increases its nuclear expression in CCA cells

**Fig. 5:**
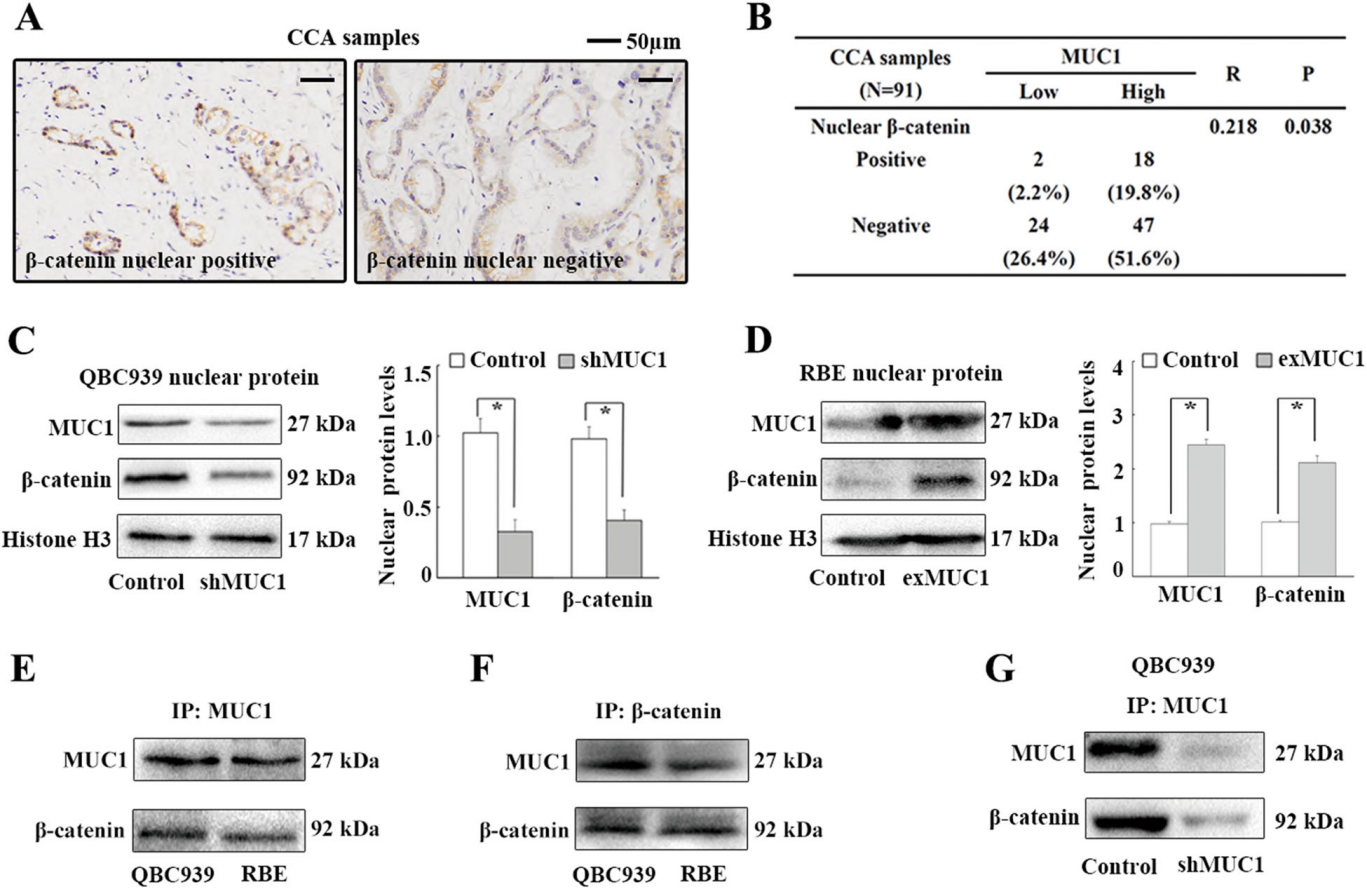
MUC1 binds to *β*-catenin and increases its nuclear expression in CCA cells. **a** IHC staining of *β*-catenin in CCA samples. **b** Correlations between MUC1 expression and nuclear *β*-catenin expression in 91 CCA samples by Spearman’s correlation analyses. **c** Impact of MUC1 knockdown on nuclear protein levels of MUC1 and *β*-catenin in QBC939 cells by WB analyses. **d** Impact of MUC1 overexpression on nuclear protein levels of MUC1 and *β*-catenin in RBE cells by WB analyses. **e**, **f** MUC1 binds to *β*-catenin in QBC939 and RBE cells by IP analyses. **g** MUC1 knockdown significantly decreased the binding of MUC1 and *β*-catenin in QBC939 cells. Control: cells transfected with empty lentivirus vectors. shMUC1: cells transfected with lentivirus vectors encoding short hairpin RNA targeting human MUC1. exMUC1: cells transfected with lentivirus vectors encoding human MUC1 CDS DNA. *P < 0.05.

The mechanism by which MUC1 promotes EMT in CCA was investigated. *β*-Catenin acts as an important transcriptional coactivator (TCA) regulating EMT in cancers and the nuclear expression of *β*-catenin is a key contributor to this process^17,25^. It has been demonstrated that MUC1 binds to *β*-catenin and increases its nuclear expression in pancreatic cancer^21,23^. Thus, we aimed to investigate whether MUC1 promoted EMT by increasing nuclear *β*-catenin expression in CCA cells. We first investigated the correlation between MUC1 and unclear *β*-catenin expression in 91 CCA samples. Twenty CCA samples (22.0%) showed nuclear expression of *β*-catenin in CCA cells (Fig. 5a) and Spearman’s correlation analyses revealed a significant positive correlation between MUC1 expression and nuclear *β*-catenin expression in CCA samples (Fig. 5b). Second, the effect of MUC1 on nuclear *β*-catenin expression was investigated in QBC939 and RBE cells. The nuclear protein levels of *β*-catenin and MUC1 were significantly reduced following MUC1 knockdown in QBC939 cells (Fig. 5c). Conversely, the nuclear protein levels of *β*-catenin and MUC1 were increased following MUC1 overexpression in RBE cells (Fig. 5d). Third, MUC1 bound to *β*-catenin in both QBC939 and RBE cells based on immunoprecipitation (IP) analyses (Fig. 5e, f). Moreover, MUC1 knockdown significantly decreased the binding of MUC1 to *β*-catenin in QBC939 cells (Fig. 5g). Together, the results suggest that MUC1 binds to *β*-catenin and increases its nuclear expression in CCA cells.

### GATA6 upregulates MUC1 and promotes metastasis in CCA cell-engrafted nude mice

**Fig. 6:**
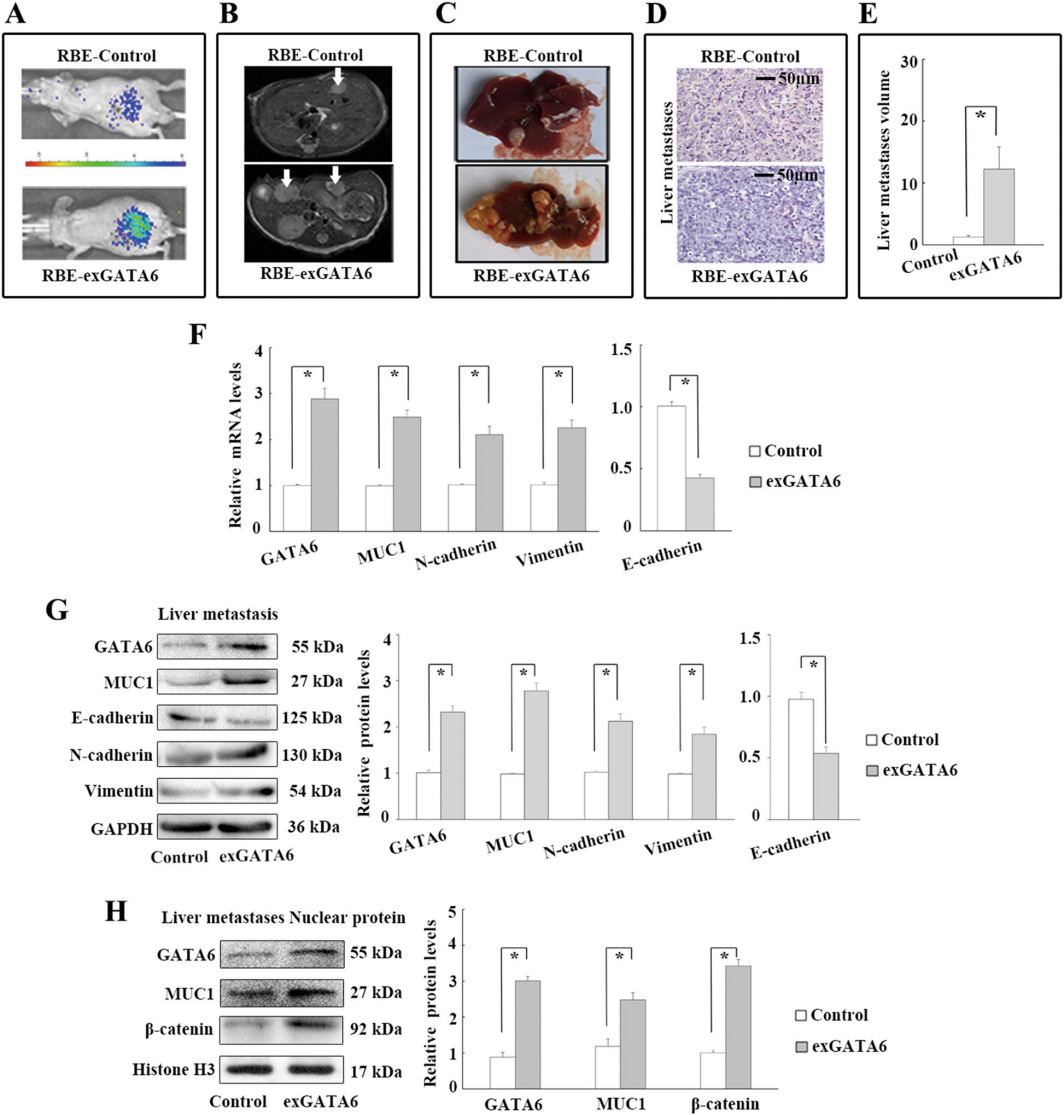
GATA6 upregulates MUC1 and promotes metastases in CCA cell spleen-engrafted nude mice. **a** Impact of GATA6 overexpression on cancer cell metastasis in RBE cell spleen-engrafted nude mice by in vivo luciferase. **b**, **c** Impact of GATA6 overexpression on liver metastasis in RBE cell spleen-engrafted nude mice by MRI and gross tissue analysis. **d** H.E. staining of liver metastases. **e** Impact of GATA6 overexpression on liver metastases volume in RBE cell spleen-engrafted nude mice. **f** Impact of GATA6 overexpression on mRNA levels of EMT markers and MUC1 in liver metastases tissues. **g** Impact of GATA6 overexpression on protein levels of EMT markers and MUC1 in liver metastases tissues. **h** Impact of GATA6 overexpression on nuclear protein levels of *β*-catenin and MUC1 in liver metastases tissues. Control: cells transfected with empty lentivirus vectors. exGATA6: cells transfected with lentivirus vectors encoding human GATA6 CDS DNA. *P < 0.05.

The role of GATA6 in MUC1 expression and CCA cell metastasis were investigated in CCA cell-engrafted nude mice. ExGATA6 and control RBE cells were injected into the spleens of nude mice. After 8 weeks, in vivo bioluminescence analyses showed a higher fluorescence intensity in exGATA6 cell-injected mice (Fig. 6a). MRI analyses showed that GATA6 overexpression increased liver metastasis (Fig. 6b). The liver metastasis volume was significantly higher in the GATA6 overexpression group than in the control group according to gross tissue analysis (Fig. 6c–e). Significantly lower mRNA and protein levels of E-cadherin and higher mRNA and protein levels of N-cadherin and vimentin were observed in exGATA6 liver metastatic tissues than in control liver metastatic tissues based on real-time PCR and WB analyses (Fig. 6f, g). In addition, the mRNA and protein levels of MUC1 were higher in exGATA6 liver metastatic tissues (Fig. 6f, g). Moreover, significantly higher nuclear protein levels of MUC1 and *β*-catenin were detected in exGATA6 liver metastatic tissues than in control liver metastatic tissues (Fig. 6h). Together, the results show that GATA6 upregulates MUC1 and promotes metastasis in CCA cell-implanted nude mice.

### GATA6 and MUC1 are significantly associated with poor prognosis of CCA patients

**Fig. 7:**
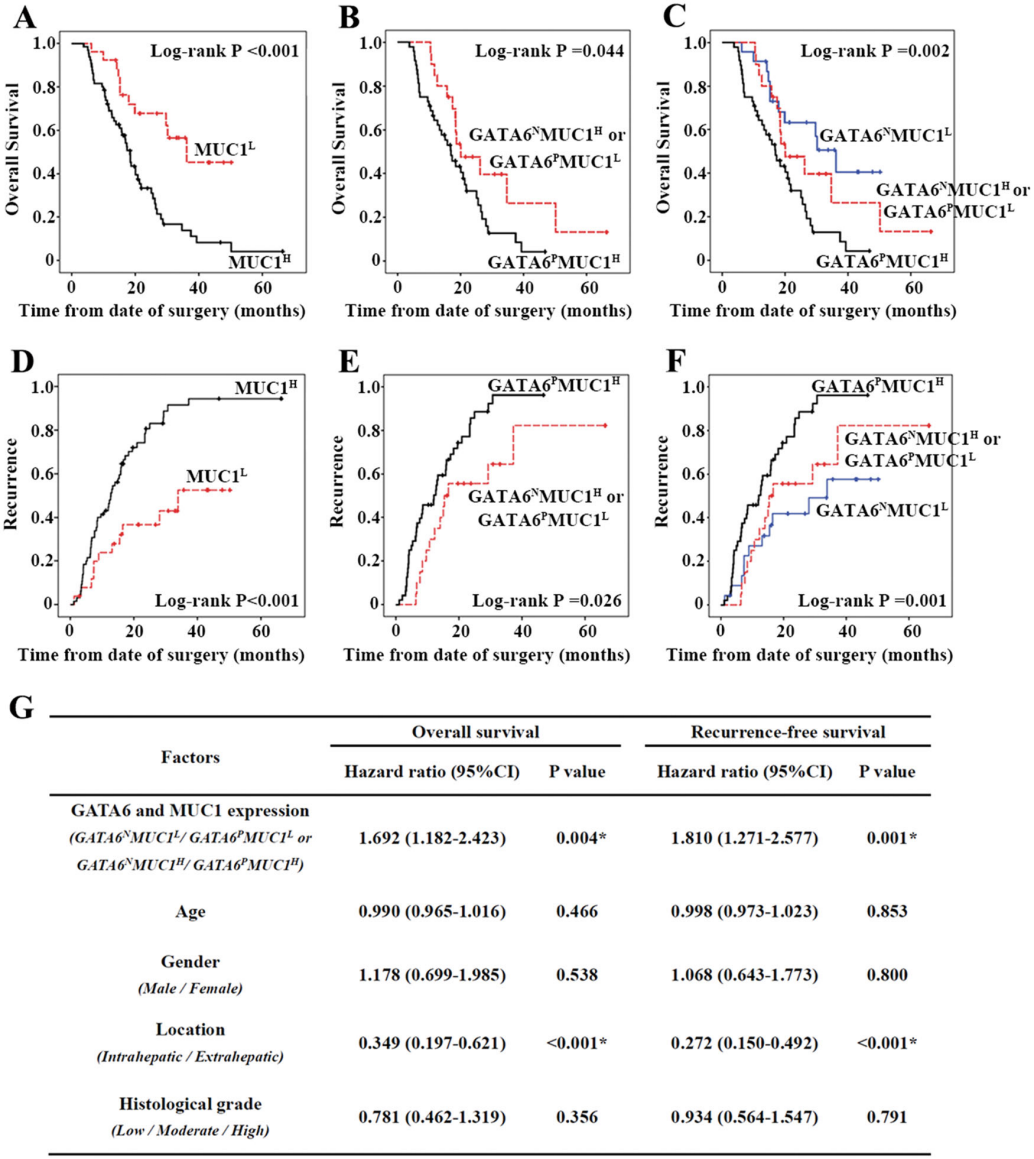
GATA6 and MUC1 are significantly associated with poor prognosis in CCA patients. **a**–**f** Expression of GATA6 and MUC1 impacts overall survival and tumour recurrence in 91 patients with CCA following surgical resection by Kaplan–Meier analysis. **g** Cox analysis for overall survival and recurrence-free survival of 91 patients with CCA following surgical resection. GATA6^p^: GATA6 positive. GATA6^N^: GATA6 negative. MUC1^H^: MUC1 high expression. MUC1^L^: MUC1 low expression. *Statistical significance.

Finally, the roles of GATA6 and MUC1 expression in prognosis were analysed in 91 patients with CCA. According to Kaplan–Meier analyses, patients with high MUC1 expression experienced a significantly shorter overall survival and earlier recurrence than patients with low MUC1 expression (Fig. 7a, d), indicating that MUC1 expression was associated with poor prognosis of CCA. Among the 91 patients, 48 showed both positive GATA6 expression and high MUC1 expression, 20 showed either positive GATA6 expression or high MUC1 expression, and 23 showed both negative GATA6 expression and low MUC1 expression. Patients with both positive GATA6 expression and high MUC1 expression experienced shorter overall survival and earlier recurrence than patients with either positive GATA6 expression or high MUC1 expression (Fig. 7b, e). In addition, patients with both negative GATA6 expression and low MUC1 expression, patients with either positive GATA6 expression or high MUC1 expression, and patients with both positive GATA6 expression and high MUC1 expression showed progressively shorter survival (Fig. 7c, f). According to the Cox analysis, the GATA6 and MUC1 co-expression profile was an independent predictor off shorter overall survival and recurrence-free survival (hazard ratio (HR) = 1.692, 95% confidence interval (95% CI) = 1.182–2.423, *P* = 0.004; HR = 1.810, 95% CI = 1.271–2.577, *P* = 0.004) (Fig. 7g). Together, the results suggest that GATA6 and MUC1 are significantly associated with poor prognosis of CCA patients.

## Discussion

Every advancement in clinical therapy is based on a wealth of basic data and an improved understanding of cancer biology. Here we expand upon prior data, indicating that GATA6 promotes EMT through a novel mechanism and show the potential of GATA6 as a future prognostic predictor and biotherapy for patients with CCA.

The roles of GATA6 in cancers are complicated by its effect in promoting or suppressing progression of different types of cancer. No clear explanation for this conundrum has been identified. We postulate that it might depend on differences in coactivator/repressor expression and epigenetic regulation. Our published and recent unpublished data indicate that GATA6 functions as an oncoprotein in CCA, contributing to cancer cell metastasis, angiogenesis and immune escape. In addition to its effects on CCA, GATA6 has been reported to enable the self-renewal of cancer stem cells and to promote tumorigenesis and metastasis in colon cancer^5,26,27^. In addition, GATA6 promotes tumour growth and development in gastric cancer^28,29^ and sustains oncogenic lineage survival in oesophageal adenocarcinoma^30^. Based on our data and those from previous studies, GATA6 promotes cancer progression and might be a potential target for anticancer biotherapy in patients with some digestive cancers, including CCA.

We provide evidences that GATA6 promotes EMT of CCA cells by binding to MUC1 promoter and upregulating its expression. To our knowledge, the present study describes a novel mechanism by which GATA6 promotes EMT in cancer. Previous studies reported that GATA6 regulates EMT by binding to slug promoter and upregulating its expression in breast cancer^6^. However, our ChIP-Seq data did not show GATA6 enrichment at the slug promoter in CCA, indicating distinct mechanisms of GATA6 in different cancers. As a putative transcription factor (TF), GATA6 has a putative DNA-binding site that recognizes the GATA motif^20^. We identified two GATA motifs (1584 and 1456) in the MUC1 promoter, both of which are essential for GATA6-mediated regulation of MUC1 transcription. In addition, GATA6 also regulates gene transcription as a TCA that is recruited by other TFs and does not directly bind to DNA^27^. Based on our data, mutation of both the 1584 and 1456 GATA motifs completely abolished the ability of GATA6 overexpression to regulate MUC1 transcription, indicating that GATA6 upregulates MUC1 as a TF but not a TCA.

MUC1 is a transmembrane mucin that is normally expressed on the luminal surfaces of ductal epithelia, including the biliary duct^24^. MUC1 has two subunits: the N terminus (MUC1-N), which contains glycosylated tandem repeats and contributes to a physically protective function, and the C terminus (MUC1-C), which contains the transmembrane part and the cytoplasmic tail that contribute to multiple signalling interactions^31,32^. In many cancers of epithelial origin, overexpression of MUC1 in cancer cells participates in regulating cancer progression, including the EMT^33^. It has been reported that MUC1 undergoes autocleavage into MUC1-N and MUC1-C, and MUC1-C accumulates in cancer cells and binds to TFs or TCAs to improve their function by increasing their nuclear translocation^21^. According to previous studies, MUC1 contains a SAGNGGSSLS motif that binds to *β*-catenin^21,34,35^. Besides, MUC1 binds to *β*-catenin and promotes its nuclear translocation in pancreatic cancers^23^. In our study, MUC1 bound to *β*-catenin and increases its nuclear expression in CCA cells. *β*-Catenin is a putative regulator of EMT in cancer cells that functions as a TCA^25^. Several studies have shown that *β*-catenin is overexpressed in CCA (as characterized by increased nuclear expression in cancer cells) and promotes EMT of CCA cells^36^. Together, our data and the aforementioned reports suggest that MUC1 promotes EMT by increasing the nuclear expression of *β*-catenin in CCA cells.

In addition to regulating EMT, MUC1 promotes cancer progression through other mechanisms. MUC1 contributes to immune escape by upregulating PD-L1 expression in breast cancer^37^. In addition, MUC1 regulates DNA methylation by upregulating the expression of DNMT1 and DNMT3b methyltransferases in human carcinoma cells^38^. MUC1 is also considered a novel metabolic master regulator of glucose and lipid metabolism to maintain cancer cell survival under nutrient-deprived conditions^39^. Based on our finding that GATA6 is an upstream regulator of MUC1, we postulate that GATA6 might regulate CCA progression through these mechanisms, which require further investigation.

In summary, our data show the following: (1) GATA6 promotes EMT by upregulating MUC1 in CCA; (2) GATA6 upregulates MUC1 through binding to both the 1584 and 1456 GATA motifs in the promoter and enhancing its transcriptional activity; and (3) MUC1 promotes EMT by binding to *β*-catenin and increasing its nuclear expression in CCA. Together, our results suggest that GATA6 promotes EMT through MUC1/*β*-catenin pathway in CCA, and these findings have potential implications for anti-metastatic therapy.

## Supplementary information

Legends of supporting figures and tables

Supplementary FigureS1

Document S1

## Data Availability

The datasets used and/or analysed during the current study are available from the corresponding author on reasonable request.

## References

[1] Rizvi S, Khan SA, Hallemeier CL, Kelley RK, Gores GJ (2018). Cholangiocarcinoma - evolving concepts and therapeutic strategies. Nat. Rev. Clin. Oncol..

[2] Zhu AX (2015). Future directions in the treatment of cholangiocarcinoma. Best. Pract. Res. Clin. Gastroenterol..

[3] Ayanbule F, Belaguli NS, Berger DH (2011). GATA factors in gastrointestinal malignancy. World J. Surg..

[4] Sulahian R (2014). An integrative analysis reveals functional targets of GATA6 transcriptional regulation in gastric cancer. Oncogene.

[5] Tsuji S (2014). The miR-363-GATA6-Lgr5 pathway is critical for colorectal tumourigenesis. Nat. Commun..

[6] Song Y (2015). GATA6 is overexpressed in breast cancer and promotes breast cancer cell epithelial-mesenchymal transition by upregulating slug expression. Exp. Mol. Pathol..

[7] Kamijo H (2018). Aberrant CD137 ligand expression induced by GATA6 overexpression promotes tumor progression in cutaneous T-cell lymphoma. Blood.

[8] Kamnasaran D, Qian B, Hawkins C, Stanford WL, Guha A (2007). GATA6 is an astrocytoma tumor suppressor gene identified by gene trapping of mouse glioma model. Proc. Natl Acad. Sci. USA.

[9] Tan HW (2019). Deregulated GATA6 modulates stem cell-like properties and metabolic phenotype in hepatocellular carcinoma. Int. J. Cancer.

[10] Kwei KA (2008). Genomic profiling identifies GATA6 as a candidate oncogene amplified in pancreatobiliary cancer. PLoS Genet..

[11] Martinelli P (2017). GATA6 regulates EMT and tumour dissemination, and is a marker of response to adjuvant chemotherapy in pancreatic cancer. Gut.

[12] Capo-chichi CD (2003). Anomalous expression of epithelial differentiation-determining GATA factors in ovarian tumorigenesis. Cancer Res..

[13] Shen W (2019). GATA6: a new predictor for prognosis in ovarian cancer. Hum. Pathol..

[14] Tian F (2013). Aberrant expression of GATA binding protein 6 correlates with poor prognosis and promotes metastasis in cholangiocarcinoma. Eur. J. Cancer.

[15] Tian F (2017). miR-124 targets GATA6 to suppress cholangiocarcinoma cell invasion and metastasis. BMC Cancer.

[16] Brabletz T, Kalluri R, Nieto MA, Weinberg RA (2018). EMT in cancer. Nat. Rev. Cancer.

[17] Vaquero J (2017). Epithelial-mesenchymal transition in cholangiocarcinoma: From clinical evidence to regulatory networks. J. Hepatol..

[18] Campbell K, Whissell G, Franch-Marro X, Batlle E, Casanova J (2011). Specific GATA factors act as conserved inducers of an endodermal-EMT. Dev. Cell..

[19] Naito S, von Eschenbach AC, Giavazzi R, Fidler IJ (1986). Growth and metastasis of tumor cells isolated from a human renal cell carcinoma implanted into different organs of nude mice. Cancer Res..

[20] Maeda M, Ohashi K, Ohashi-Kobayashi A (2005). Further extension of mammalian GATA-6. Dev. Growth Differ..

[21] Rajabi H, Kufe D (2017). MUC1-C oncoprotein integrates a program of EMT, epigenetic reprogramming and immune evasion in human carcinomas. Biochim. Biophys. Acta.

[22] Gnemmi V (2014). MUC1 drives epithelial-mesenchymal transition in renal carcinoma through Wnt/beta-catenin pathway and interaction with SNAIL promoter. Cancer Lett..

[23] Roy LD (2011). MUC1 enhances invasiveness of pancreatic cancer cells by inducing epithelial to mesenchymal transition. Oncogene.

[24] Sasaki M, Ikeda H, Nakanuma Y (2007). Expression profiles of MUC mucins and trefoil factor family (TFF) peptides in the intrahepatic biliary system: physiological distribution and pathological significance. Prog. Histochem. Cytochem..

[25] Valenta T, Hausmann G, Basler K (2012). The many faces and functions of beta-catenin. EMBO J..

[26] Whissell G (2014). The transcription factor GATA6 enables self-renewal of colon adenoma stem cells by repressing BMP gene expression. Nat. Cell Biol..

[27] Belaguli NS (2010). GATA6 promotes colon cancer cell invasion by regulating urokinase plasminogen activator gene expression. Neoplasia.

[28] Song SH (2018). Aberrant GATA2 epigenetic dysregulation induces a GATA2/GATA6 switch in human gastric cancer. Oncogene.

[29] Chia NY (2015). Regulatory crosstalk between lineage-survival oncogenes KLF5, GATA4 and GATA6 cooperatively promotes gastric cancer development. Gut.

[30] Lin L (2012). Activation of GATA binding protein 6 (GATA6) sustains oncogenic lineage-survival in esophageal adenocarcinoma. Proc. Natl Acad. Sci. USA.

[31] Lan MS, Batra SK, Qi WN, Metzgar RS, Hollingsworth MA (1990). Cloning and sequencing of a human pancreatic tumor mucin cDNA. J. Biol. Chem..

[32] Singh PK, Hollingsworth MA (2006). Cell surface-associated mucins in signal transduction. Trends Cell Biol..

[33] Nath S, Mukherjee P (2014). MUC1: a multifaceted oncoprotein with a key role in cancer progression. Trends Mol. Med..

[34] Yamamoto M, Bharti A, Li Y, Kufe D (1997). Interaction of the DF3/MUC1 breast carcinoma-associated antigen and beta-catenin in cell adhesion. J. Biol. Chem..

[35] Wen Y, Caffrey TC, Wheelock MJ, Johnson KR, Hollingsworth MA (2003). Nuclear association of the cytoplasmic tail of MUC1 and beta-catenin. J. Biol. Chem..

[36] Zhang J, Han C, Wu T (2012). MicroRNA-26a promotes cholangiocarcinoma growth by activating beta-catenin. Gastroenterology.

[37] Maeda T (2018). MUC1-C induces PD-L1 and immune evasion in triple-negative breast cancer. Cancer Res..

[38] Rajabi H (2016). DNA methylation by DNMT1 and DNMT3b methyltransferases is driven by the MUC1-C oncoprotein in human carcinoma cells. Oncogene.

[39] Mehla K, Singh PK (2014). MUC1: a novel metabolic master regulator. Biochim. Biophys. Acta.

