# Supplementary material for: GATA6 promotes epithelial-mesenchymal transition and metastasis through MUC1/β-catenin pathway in cholangiocarcinoma
Source: Cell Death Dis. 2020 Oct 15;11(10):860. doi: 10.1038/s41419-020-03070-z (PMC7567063; doi:10.1038/s41419-020-03070-z)
Supplement: Supplementary file 1 — Legends of supporting figures and tables [file 41419_2020_3070_MOESM1_ESM.doc]

**Legends of supporting figures and tables**

**Figure S1** Aberrant expression of GATA6 is associated with poor prognosis in CCA. (A-D) IHC staining of GATA6 in CCA cancerous samples and paracancerous samples. (E) Expression of GATA6 impacts overall survival and tumor recurrence in 91 cholangiocarcinoma patients following surgical resection by Kaplan-Meier analysis.

**Table S1 Primers used in the present study.**

| **Gene** | **Primers** | **Product** |
| --- | --- | --- |
| GATA6 | GTGCCCAGACCACTTGCTAT | 106 |
|  | TGGAATTATTGCTATTACCAGAGC |  |
| MUC1 | TTTCCAGCCCGGGATACCTA | 136 |
|  | AGAGGCTGCTGCCACCATTA |  |
| E-Cadherin | AACAGGATGGCTGAAGGTGA | 192 |
|  | CCTTCCATGACAGACCCCTT |  |
| N-Cadherin | ATATTTCCATCCTGCGCGTG | 195 |
|  | GTTTGGCCTGGCGTTCTTTA |  |
| Vimentin | GAGAGGAAGCCGAAAACACC | 167 |
|  | TTCCTGAATCTGAGCCTGCA |  |
| GAPDH | AGAAGGCTGGGGCTCATTTG | 258 |
|  | AGGGGCCATCCACAGTCTTC |  |
| ChIP PCR  MUC1 promoter | GACTGCGGGACCCAAGTT | 200 |
|  | TGACAGAATGGGATTTACGG |  |

**Table S2** Correlations between clinical features and GATA6, MUC1 expression in CCA patients (N=91).

| Features | GATA6 expression | | P value*^a^* | MUC1 expression | | P value*^a^* |
| --- | --- | --- | --- | --- | --- | --- |
|  | High (51) | Low (40) |  | High (65) | Low (26) |  |
| Age | 56.8±10.2 | 55.5±9.4 | 0.537 | 56.4±9.7 | 55.7±10.3 | 0.776 |
| Gender |  |  | 0.375 |  |  | 0.550 |
| Male | 29 (56.9%) | 19 (47.5%) |  | 33 (50.8%) | 15 (57.7%) |  |
| Female | 22 (43.1%) | 21 (52.5%) |  | 32 (49.2%) | 11 (42.3%) |  |
| Location |  |  | 0.996 |  |  | 0.102 |
| Intrahepatic | 14 (27.5%) | 11 (27.5%) |  | 21 (32.3%) | 4 (15.4%) |  |
| Extrahepatic | 37 (72.5%) | 29 (72.5%) |  | 44 (67.7%) | 22 (84.6%) |  |
| Histological Grade |  |  | 0.140 |  |  | <0.001*^b^* |
| G1 | 3 (13.7%) | 6 (10.0%) |  | 2 (3.1%) | 7 (26.9%) |  |
| G2 | 35 (68.6%) | 29 (72.5%) |  | 46 (70.8%) | 18 (69.2%) |  |
| G3 | 13 (17.6%) | 5 (17.5%) |  | 17 (26.2%) | 1 (3.8%) |  |
| T classification |  |  | 0.215 |  |  | 0.332 |
| T1 | 5 (9.8%) | 2 (5.0%) |  | 5 (7.7%) | 2 (7.7%) |  |
| T2 | 33 (64.7%) | 29 (72.5%) |  | 43 (66.2%) | 15 (57.7%) |  |
| T3 | 10 (19.6%) | 9 (22.5%) |  | 14 (21.5%) | 9 (34.6%) |  |
| T4 | 3 (5.9%) | 0 (0.0%) |  | 3 (4.6%) | 0 (0.0%) |  |
| Lymph-node metastasis |  |  | 0.024*^b^* |  |  | 0.010*^b^* |
| Yes | 30 (58.8%) | 14 (35.0%) |  | 37 (56.9%) | 7 (26.9%) |  |
| No | 21 (41.2%) | 26 (65.0%) |  | 28 (43.1%) | 19 (73.1%) |  |
| Distant metastasis |  |  | >1.000 |  |  | >1.000 |
| Yes | 0 (0.0%) | 0 (0.0%) |  | 0 (0.0%) | 0 (0.0%) |  |
| No | 51 (100.0%) | 40 (100.0%) |  | 65 (100.0%) | 26 (100.0%) |  |

*a*: P value is for t test (continuous variables) or chi-square or Fisherˊs exact test (catigorical variables).

*b*: P<0.05, statistical significance.

**Table S3** ChIP-sequencing data

| **Symbol** | **Chr** | **Length** | **-10*LOG(p-value)** | **Fold_enrichment** | **FDR(%)** |
| --- | --- | --- | --- | --- | --- |
| **CYP2A6** | chr19 | 357 | 3100 | 487.8 | 0 |
| **C8orf31** | chr8 | 400 | 3100 | 190 | 0 |
| **C21orf62-AS1** | chr21 | 372 | 3100 | 163.68 | 0 |
| **CRYBB2** | chr22 | 488 | 3100 | 158.33 | 0 |
| **HAUS3** | chr4 | 689 | 3100 | 140 | 0 |
| **POLN** | chr4 | 689 | 3100 | 140 | 0 |
| **PDE1C** | chr7 | 357 | 3100 | 134.38 | 0 |
| **NGB** | chr14 | 389 | 3100 | 132.26 | 0 |
| **LOC101927523** | chr10 | 353 | 3100 | 95.83 | 0 |
| **KCNIP4** | chr4 | 352 | 3100 | 88.1 | 0 |
| **ZMYM3** | chrX | 324 | 3100 | 84.21 | 0 |
| **HAR1B** | chr20 | 536 | 3100 | 81.82 | 0 |
| **CASC4** | chr15 | 427 | 3100 | 79 | 0 |
| **MDGA1** | chr6 | 342 | 3100 | 77.27 | 0 |
| **MIR6766** | chr15 | 725 | 3100 | 64.17 | 0 |
| **SLITRK3** | chr3 | 339 | 3100 | 61.11 | 0 |
| **RHOC** | chr1 | 329 | 3100 | 58.33 | 0 |
| **COL6A1** | chr21 | 796 | 3100 | 57.31 | 0 |
| **TLR6** | chr4 | 316 | 3100 | 56.82 | 0 |
| **LOC643923** | chr11 | 335 | 3100 | 54.44 | 0 |
| **NR2F1** | chr5 | 523 | 3100 | 54.17 | 0 |
| **LDB2** | chr4 | 566 | 2728.66 | 50 | 0 |
| **RHOBTB2** | chr8 | 311 | 1485.5 | 50 | 0.19 |
| **CPLX2** | chr5 | 353 | 3100 | 38.33 | 0 |
| **BAIAP2L2** | chr22 | 365 | 3100 | 38.02 | 0 |
| **DDX11L1** | chr1 | 842 | 3100 | 37.12 | 0 |
| **DHRS9** | chr2 | 348 | 3100 | 35.48 | 0 |
| **TMX4** | chr20 | 419 | 2855.09 | 29.41 | 0 |
| **RNASET2** | chr6 | 454 | 3100 | 28.38 | 0 |
| **CCDC94** | chr19 | 326 | 3100 | 27.71 | 0 |
| **BRWD3** | chrX | 318 | 2798.38 | 25.44 | 0 |
| **LOC100132111** | chr1 | 646 | 1889.89 | 25 | 0 |
| **MIR4697HG** | chr11 | 337 | 824.53 | 25 | 0.08 |
| **MIR6511A1** | chr16 | 434 | 685.08 | 25 | 0.06 |
| **MIR6511A2** | chr16 | 434 | 685.08 | 25 | 0.06 |
| **MIR6511A3** | chr16 | 434 | 685.08 | 25 | 0.06 |
| **MIR6511A4** | chr16 | 434 | 685.08 | 25 | 0.06 |
| **TARBP2** | chr12 | 308 | 3100 | 24.64 | 0 |
| **HOXB13** | chr17 | 485 | 1993.75 | 24.19 | 0 |
| **STX6** | chr1 | 424 | 3100 | 21.91 | 0 |
| **CABIN1** | chr22 | 222 | 2619.57 | 21.74 | 0 |
| **LRP2** | chr2 | 637 | 3100 | 21.34 | 0 |
| **PLA2G7** | chr6 | 317 | 1849.11 | 21.15 | 0 |
| **RYR3** | chr15 | 330 | 1120.94 | 20.59 | 0.13 |
| **ADM2** | chr22 | 320 | 977.88 | 18.75 | 0.1 |
| **DIO3OS** | chr14 | 372 | 1319.57 | 18.6 | 0.17 |
| **TYR** | chr11 | 807 | 3100 | 18.5 | 0 |
| **CMSS1** | chr3 | 323 | 3100 | 17.76 | 0 |
| **ALDH3A1** | chr17 | 331 | 1486.47 | 16.67 | 0.19 |
| **IL33** | chr9 | 530 | 862.01 | 15.2 | 0.06 |
| **CACNA1C-AS1** | chr12 | 369 | 769.38 | 15 | 0.07 |
| **RUFY3** | chr4 | 321 | 1030.06 | 13.46 | 0.11 |
| **ESD** | chr13 | 202 | 1289.56 | 12.5 | 0.16 |
| **LOC101928075** | chr14 | 204 | 1269.51 | 12.5 | 0.16 |
| **MCTP2** | chr15 | 375 | 931.22 | 12.5 | 0.09 |
| **MIR6836** | chr7 | 209 | 1249.51 | 12.5 | 0.15 |
| **SCNN1G** | chr16 | 278 | 526.31 | 12.5 | 0.08 |
| **SIGLEC5** | chr19 | 526 | 611.37 | 11.9 | 0.05 |
| **SLC4A1** | chr17 | 278 | 698.67 | 11.9 | 0.08 |
| **CEP83-AS1** | chr12 | 517 | 521.95 | 11.76 | 0.04 |
| **MUC1** | chr1 | 468 | 849.68 | 11.62 | 0.04 |
| **CST9** | chr20 | 338 | 778.27 | 11.54 | 0.07 |
| **CHRM3-AS2** | chr1 | 208 | 1217.15 | 11.36 | 0.15 |
| **SAMD13** | chr1 | 322 | 957.73 | 11.27 | 0.1 |
| **DBH-AS1** | chr9 | 405 | 1253.33 | 11.14 | 0.15 |
| **PIWIL1** | chr12 | 494 | 656.62 | 11.11 | 0.06 |
| **C2orf15** | chr2 | 593 | 711.36 | 10.87 | 0.06 |
| **ZNF41** | chrX | 289 | 790.64 | 10.87 | 0.07 |
| **DKFZp434J0226** | chr19 | 412 | 683.28 | 10.53 | 0.06 |
| **SHOX** | chrY | 803 | 1570.21 | 10.04 | 0.2 |
| **IGFL2** | chr19 | 319 | 1066.26 | 10 | 0.12 |
| **SP140** | chr2 | 572 | 779.92 | 10 | 0.07 |
| **PDCD2** | chr6 | 263 | 1029.19 | 9.68 | 0.11 |
| **ANPEP** | chr15 | 270 | 831.11 | 9.62 | 0.08 |
| **ATP6V0A2** | chr12 | 450 | 643.9 | 9.52 | 0.05 |
| **FLJ42393** | chr3 | 493 | 976.45 | 9.52 | 0.1 |
| **LOC101927914** | chr7 | 316 | 796.92 | 9.48 | 0.07 |
| **SOX5** | chr12 | 338 | 1204.7 | 9.26 | 0.14 |
| **UPK1A** | chr19 | 367 | 550.32 | 9.26 | 0.04 |
| **MIR3675** | chr1 | 790 | 651.9 | 9.09 | 0.06 |
| **MTMR2** | chr11 | 352 | 686.63 | 9.09 | 0.06 |
| **USP6** | chr17 | 203 | 1172.57 | 8.93 | 0.14 |
| **NAV3** | chr12 | 320 | 1404.57 | 8.8 | 0.18 |
| **WDR74** | chr11 | 389 | 687.82 | 8.75 | 0.06 |
| **PXDC1** | chr6 | 371 | 520.81 | 8.62 | 0.04 |
| **MECOM** | chr3 | 332 | 721.18 | 8.57 | 0.06 |
| **DDI2** | chr1 | 373 | 514.5 | 8.33 | 0.04 |
| **DHCR7** | chr11 | 522 | 510.32 | 8.33 | 0.04 |
| **GTF2IRD2** | chr7 | 318 | 762.95 | 8.33 | 0.07 |
| **GTF2IRD2B** | chr7 | 318 | 762.95 | 8.33 | 0.07 |
| **PEG3-AS1** | chr19 | 229 | 986 | 8.33 | 0.1 |
| **MMP19** | chr12 | 588 | 823.22 | 8 | 0.08 |
| **ACMSD** | chr2 | 531 | 737.23 | 7.96 | 0.07 |
| **C7orf25** | chr7 | 392 | 560.38 | 7.89 | 0.04 |
| **ECSCR** | chr5 | 432 | 533.89 | 7.89 | 0.04 |
| **ODF2** | chr9 | 381 | 590.98 | 7.89 | 0.05 |
| **GOLGA6L6** | chr15 | 314 | 629.83 | 7.85 | 0.05 |
| **NCF2** | chr1 | 539 | 653.77 | 7.81 | 0.06 |
| **ZNF462** | chr9 | 316 | 628.77 | 7.81 | 0.05 |
| **PDZK1IP1** | chr1 | 313 | 877.86 | 7.78 | 0.08 |
| **MACC1-AS1** | chr7 | 303 | 790.3 | 7.69 | 0.07 |
| **DDX6** | chr11 | 391 | 466.43 | 7.58 | 0.03 |
| **HIST2H2BA** | chr1 | 416 | 709.76 | 7.58 | 0.06 |
| **GDF5** | chr20 | 206 | 1310.89 | 7.5 | 0.16 |
| **PRRC2C** | chr1 | 205 | 1310.89 | 7.5 | 0.16 |
| **ZSWIM8** | chr10 | 649 | 649.9 | 7.41 | 0.05 |
| **CASC3** | chr17 | 297 | 792.58 | 7.32 | 0.07 |
| **MFF** | chr2 | 332 | 681.66 | 7.32 | 0.06 |
| **C1orf198** | chr1 | 214 | 1025.1 | 7.14 | 0.11 |
| **CHGB** | chr20 | 625 | 661.96 | 7.14 | 0.06 |
| **KCNH2** | chr7 | 306 | 640.69 | 7.14 | 0.05 |
| **OGDH** | chr7 | 364 | 599.13 | 7.14 | 0.05 |
| **TXNRD3** | chr3 | 346 | 546.75 | 7.14 | 0.04 |
| **DDI1** | chr11 | 203 | 1298.76 | 6.98 | 0.16 |
| **KRTAP5-2** | chr11 | 347 | 541.05 | 6.94 | 0.04 |
| **NR2C1** | chr12 | 416 | 593.04 | 6.9 | 0.05 |
| **NAE1** | chr16 | 284 | 965.58 | 6.86 | 0.1 |
| **C16orf45** | chr16 | 683 | 730.5 | 6.67 | 0.06 |
| **PRAMEF1** | chr1 | 528 | 420.06 | 6.67 | 0.03 |
| **PTPN3** | chr9 | 551 | 527.92 | 6.67 | 0.04 |
| **SLC17A7** | chr19 | 452 | 446.76 | 6.67 | 0.03 |
| **SNORD48** | chr6 | 553 | 527.17 | 6.67 | 0.04 |
| **ZNF221** | chr19 | 559 | 518.21 | 6.45 | 0.04 |
| **DLGAP1-AS1** | chr18 | 219 | 955.79 | 6.41 | 0.1 |
| **MAP3K14** | chr17 | 537 | 455.53 | 6.25 | 0.03 |
| **PRDM9** | chr5 | 483 | 626.07 | 6.25 | 0.05 |
| **RBP5** | chr12 | 372 | 461.22 | 6.25 | 0.03 |
| **WTAPP1** | chr11 | 203 | 1049.16 | 6.25 | 0.11 |
| **CD300LD** | chr17 | 399 | 502.31 | 5.99 | 0.04 |
| **SLCO4A1** | chr20 | 319 | 569.06 | 5.95 | 0.04 |
| **IL34** | chr16 | 420 | 437.86 | 5.88 | 0.03 |
| **KCNN3** | chr1 | 522 | 513.31 | 5.88 | 0.04 |
| **SNX16** | chr8 | 474 | 3100 | 5.88 | 0 |
| **GRTP1** | chr13 | 584 | 617.65 | 5.84 | 0.05 |
| **PCDHB2** | chr5 | 391 | 419.85 | 5.81 | 0.03 |
| **CKMT1A** | chr15 | 534 | 442.7 | 5.77 | 0.03 |
| **LAMB4** | chr7 | 247 | 776.06 | 5.43 | 0.07 |
| **WNT2** | chr7 | 474 | 515.75 | 5.41 | 0.04 |
| **NECAP2** | chr1 | 583 | 414.9 | 5.36 | 0.03 |
| **RPSA** | chr3 | 550 | 372.84 | 5.26 | 0.02 |
| **STYK1** | chr12 | 526 | 488.85 | 5.26 | 0.04 |
| **TRAF5** | chr1 | 487 | 393.49 | 5.26 | 0.03 |
| **ALPK1** | chr4 | 421 | 464.88 | 5.17 | 0.03 |
| **SDCBP2** | chr20 | 338 | 576.79 | 5.07 | 0.05 |
| **AKR1E2** | chr10 | 548 | 413.69 | 5 | 0.03 |
| **ATP12A** | chr13 | 564 | 408.77 | 5 | 0.03 |
| **BSX** | chr11 | 587 | 401.95 | 5 | 0.03 |
| **CACNA2D4** | chr12 | 409 | 530.05 | 5 | 0.04 |
| **CLCA1** | chr1 | 243 | 909.96 | 5 | 0.09 |
| **DUSP5P1** | chr1 | 312 | 643.7 | 5 | 0.05 |
| **HDAC6** | chrX | 277 | 632.35 | 5 | 0.05 |
| **LINC01524** | chr20 | 367 | 433.81 | 5 | 0.03 |
| **LOC100505878** | chr5 | 512 | 425.33 | 5 | 0.03 |
| **LPPR4** | chr1 | 554 | 362.95 | 5 | 0.02 |
| **MIR7641-2** | chr1 | 613 | 528.22 | 5 | 0.04 |
| **RCCD1** | chr15 | 466 | 392.26 | 5 | 0.03 |
| **VTRNA1-3** | chr5 | 515 | 375.29 | 5 | 0.02 |
