# Supplementary figures and images for: GATA6 promotes epithelial-mesenchymal transition and metastasis through MUC1/β-catenin pathway in cholangiocarcinoma
Source: Cell Death Dis. 2020 Oct 15;11(10):860. doi: 10.1038/s41419-020-03070-z (PMC7567063; doi:10.1038/s41419-020-03070-z)

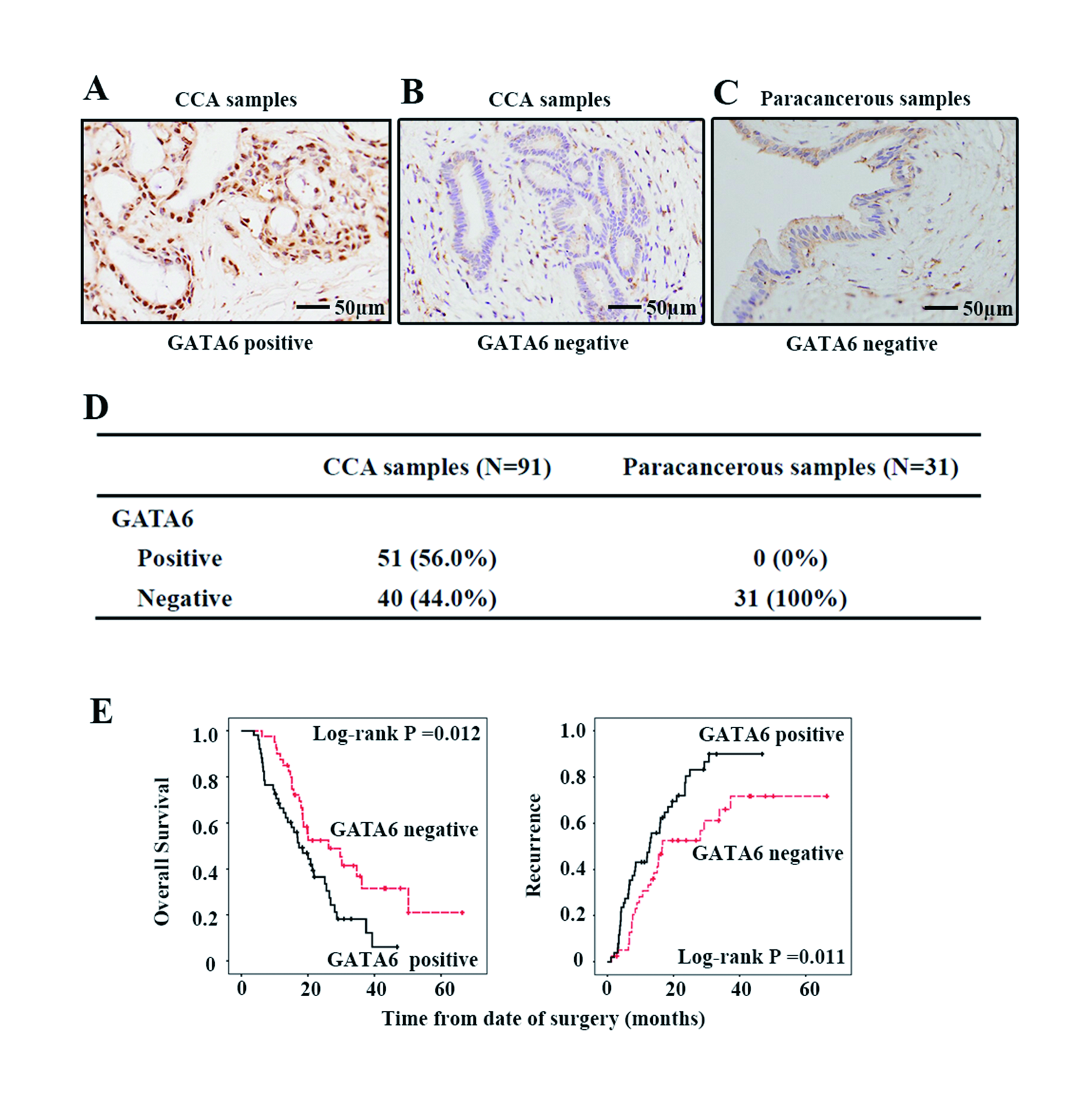

Supplement: Supplementary file 2 — Supplementary FigureS1 [file 41419_2020_3070_MOESM2_ESM.tif]
