# Supplementary material for: GATA6 promotes epithelial-mesenchymal transition and metastasis through MUC1/β-catenin pathway in cholangiocarcinoma
Source: Cell Death Dis. 2020 Oct 15;11(10):860. doi: 10.1038/s41419-020-03070-z (PMC7567063; doi:10.1038/s41419-020-03070-z)
Supplement: Supplementary file 3 — Document S1 [file 41419_2020_3070_MOESM3_ESM.doc]

**Document S1 (Methods)**

**Real-time PCR**

Total RNA was extracted from CCA cells using RNAiso Plus (Takara, Beijing, China). The cDNA synthesis was performed using the SYBR Exscript RT-PCR Kit (Takara, Beijing, China). Quantitative PCR was performed using the SYBR Premix Ex Taq^TM^ II Kit (Takara, Beijing, China). PCR reaction conditions for all assays were 94℃ for 30 seconds, followed by 40 cycles of amplification (95℃ for 5 seconds, 60℃ for 30 seconds and 72℃ for 30 seconds). GAPDH mRNA was used to normalize RNA inputs. Primer sequences were listed in Table S1. Experiments were repeated independently three times

**Western-blot (WB)**

Total proteins or nuclear protein was isolated using RIPA Lysis Buffer (Beyotime, Shanghai, China) or Nuclear Protein Extraction Kit (Beyotime, Shanghai, China), respectively. For immunoblotting, equal amounts of proteins were separated on 5%-8% SDS-PAGE and electrophoretically transferred onto nitrocellulose membranes (Millipore, MA, USA). After blocking in TBST containing 5% milk for 2 hours at RT, the membrane were blotted with antibodies overnight at 4°C: GATA6 (1:1000, Cell Signaling Technology, Danvers, MA, USA), MUC1 (1:1000, Abcam, Cambridge, MA, USA), E-Cadherin (1:2000, Proteintech, Wuhan, China), N-Cadherin (1:2000, Proteintech, Wuhan, China), vimentin (1:2000, Proteintech, Wuhan, China), GAPDH (1:2000, Proteintech, Wuhan, China), β-catenin (1:1000, Proteintech, Wuhan, China) or Histone H3 (1:500, Cell Signaling Technology, Danvers, MA, USA). After being washed with TBST and incubated with secondary antibody (1:3000, Proteintech, Wuhan, China) for 2h at room temperature, immunocomplexes were visualized using the chemiluminesence (GE, MA, USA). Experiments were repeated independently three times.

**Wound-healing analyses (Migration)**

Cells were seeded in a 6-well plate, grown until confluence, and then starved overnight. A linear wound was made by scraping a pipette tip, and the cells were cultured with FBS-free 1640 medium. The cell motility in terms of wound closure was measured by photographing at three random fields 72 hours after wounding. Experiments were repeated independently three times.

**Transwell analyses (Invasion)**

2×10^5^ cells were suspended in 400 µL FBS-free 1640 medium and seeded in the top chamber that had been coated with a layer of extracellular matrix (BD Biosciences, CA, USA). The complete medium with 10% FBS (500 µl) was added to the bottom chamber. After 48 h of incubation, the cells which had invaded through the extracellular matrix layer to the lower surface of the filters were stained. Photographs of three randomly selected fields of the fixed cells were captured, and cells were counted. Experiments were repeated independently three times.
